# Six MicroRNA Prognostic Models for Overall Survival of Lung Adenocarcinoma

**DOI:** 10.1155/2022/5955052

**Published:** 2022-08-27

**Authors:** Juan Li, Xuyu Gu, Chanchan Gao, Jun Zhang

**Affiliations:** ^1^Department of Respiratory Medicine, Zhongda Hospital, School of Medicine, Southeast University, Nanjing 21009, China; ^2^Department of Oncology, Zhongda Hospital, School of Medicine, Southeast University, Nanjing 210009, China; ^3^Department of Respiratory Medicine, The Fourth Affiliated Hospital of Nanjing Medical University, Nanjing 210031, China

## Abstract

**Objective:**

The purpose of this study is to screen for microRNAs (miRNAs) associated with the prognosis of lung adenocarcinoma (LUAD) and to explore its prognosis and effects on the tumor microenvironment in patients with LUAD.

**Methods:**

Gene expression data, miRNA expression data, and clinical data for two different databases, TCGA-LUAD and CPTAC-3 LUAD, were downloaded from the GDC database. The miRNA prognosis of LUAD was filtered by the Cox proportional hazard model and the Least Absolute Shrinkage and Selection Operator (LASSO) regression model. The performance of the model was validated by time-dependent receiver operating characteristics (ROC) curves. Possible biological processes associated with the miRNAs target gene were analyzed through Gene Ontology (GO) and Kyoto Encyclopedia of Genes and Genomes (KEGG). Finally, the prognostic model was scored by risk, divided into high- and low-risk groups by median, and the differences in the immersion level of 21 immune cells in the high- and low-risk groups were assessed. To gain a deeper understanding of the underlying mechanism behind the model, the two most important miRNAs in the model, miR-195-3p and miR-5571-5p, were selected for HPA database validation and ceRNA network construction.

**Results:**

Of the 209 variance expressions identified in the screening analysis, 145 were upregulated and 64 were downregulated by miRNAs. The prognostic models of six miRNA genes were obtained: miR-195-3p, miR-5571-5p, miR-584-3p, miR-494-3p, miR-4664-3p, and miR-1293. These six genes were significantly associated with survival rates in LUAD patients. In particular, miR-1293, miR-195-3p, and miR-5571-5p are highly correlated with OS. The higher expression of miR-195-3p and miR-5571-5p, the better survival of LUAD OS is, and these two miRNA expressions contribute the most to the model. Finally, after sorting the risk scores calculated from low to high using the prognostic model, the patients with higher scores had shorter survival time and higher frequency of death, and there were significant differences in the immersion levels of 21 immune cells in the high- and low-risk groups. ceRNA network analysis found that TM9SF3 was regulated by miR-195-3p and was highly expressed in the tissues of LUAD patients, and the prognosis of the patients was poor.

**Conclusions:**

miR-195-3p, miR-5571-5p, miR-584-3p, miR-494-3p, miR-4664-3p, and miR-1293 may be used as new biomarkers for prognosis prediction of LUAD. Our results also identified a lncRNA MEG3/miR-195-3p/RAB1A/TM9SF3 regulatory axis, which may also play an important role in the progression of LUAD. Further study needs to be conducted to verify this result.

## 1. Introduction

Lung cancer is a major cause of cancer-related mortalities all over the world as well as the most frequent form of cancer in men, and the second most frequent in women [1]. Almost 4/5 of all types of lung cancer are non-small cell lung cancer (NSCLC), and lung adenocarcinoma (LUAD) being the most common NSCLC histological subtype [2]. Despite improvements in molecular diagnosis and therapy, the prognosis of LUAD remains poor and the risk of metastasis and recurrence remains high [3]. Most LUAD patients are identified at a late stage due to a lack of adequate diagnostic techniques, and the 5-year survival rate of patients is identified as poor (approximately 17.4%) [4]. Patients with lung cancer, who receive early surgical resection, have a 5-year survival rate of up to 70% [5]. Hence, it is important to identify the biomarkers and potential therapeutics for the diagnosis and prognosis of LUAD.

MicroRNAs (miRNAs) are a kind of noncoding RNA that has about 19–25 nucleotides and regulates the expression of genes after transcription [6]. It is found to be improperly expressed in many malignancies (including lung cancer) and can be utilized as oncogenes or tumor suppressor genes [7]. There is substantial evidence that miRNAs regulate carcinogenesis processes including cell maturity, growth, invasion, autophagy, motility, invasion, and apoptosis [8, 9]. Therefore, miRNA has great potential as a promising marker for diagnosis, prognosis, and personalized targeted therapy. However, in LUAD, the function of miRNA in the prognosis of cancer patients and tumor microenvironment has not been well elucidated. Therefore, disease management and treatment need to establish a lung cancer risk prediction model by screening biomarkers for lung cancer progression.

**Figure 1:**
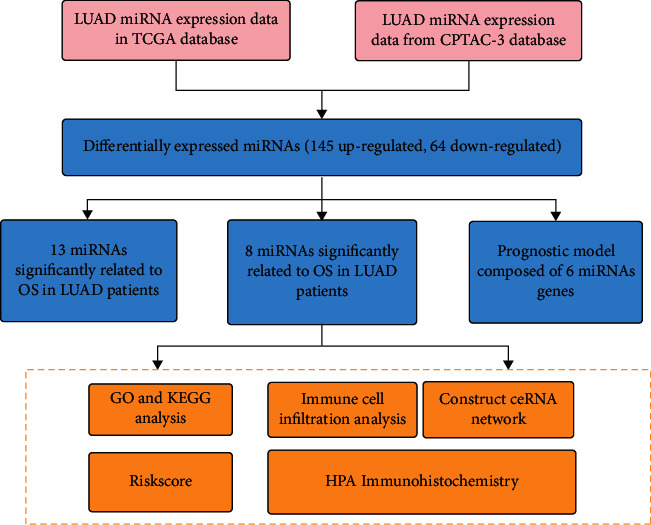
Schematic of prognostic model workflow for the overall survival of six miRNAs.

Gene expression data, miRNA expression data, and medical information for two different projects, TCGA-LUAD, and CPTAC-3 LUAD, were downloaded from the GDC database. Firstly, the intersection of differential expression of miRNAs in tumor tissues and healthy tissues was screened out in both the data sets, and then univariate Cox regression detected the prognosis-related miRNAs. The prognostic model consisting of six genes (miR-195-3p, miR-5571-5p, miR-584-3p, miR-494-3p, miR-4664-3p, and miR-1293) was obtained and constructed using LASSO. The target genes of the miRNAs were then identified and analyzed using the Gene Ontology (GO) function and Kyoto Encyclopedia of Genes and Genomes (KEGG) pathway enrichment analysis. The relationship of risk score with immune cell infiltration was examined to understand the model's mechanism. The detailed workflow diagram is shown in Figure 1.

## 2. Materials and Methods

### 2.1. Acquisition of Data

The GDC (Global Data Cente) database (https://portal.gdc.cancer.gov/) is the largest collection of cancer gene information that stores data like gene expression, DNA methylation, miRNA expression data, SNP, copy number variation, and others. There are currently two publicly available LUAD tumor data in the GDC data. Among them, TCGA-LUAD (normal group: 45, tumor group: 513) and CPTAC-3 (normal group: 197, tumor group: 219) included miRNA expression data from LUAD patients with sufficient sample size. The level 3 miRNA transcript expression data of these two projects was downloaded and compiled into an arm-level expression matrix. We used TCGA biolinks to download clinical data and post-expression data standardized by FPKM (FragmentsPer Kilobase per Million) at level 3. The FPKM data eliminated the effects of library construction and sequencing depth. In the following analysis, the expression levels of the samples may be directly compared.

### 2.2. Construction of Model and Prognosis

R software package DESeq2 was utilized to explore the differential expression of TCGA and CPTAC-3, and obtained 209 genes (in tumor tissues upregulated: 145, downregulated: 64) with significant differences (*p* adj< 0.05 and |log2FoldChange| > 1) in expression in normal tissues and tumor tissues by taking the intersection. A univariate Cox regression analysis of significantly differentially expressed miRNA genes was done, and 13 miRNA genes were shown to have a significant connection to overall survival (OS) in LUAD patients (*p*-value ≤ 0.05). At the same time, to obtain the genes most likely to be related to survival, we used the KM test to further screen. The samples were grouped as high-expression and low-expression categories, with the median serving as the cutoff. By analyzing the impact of each miRNA expression on OS, 8 miRNAs with a high correlation to OS prognosis (*p*-value < 0.05) were obtained. Furthermore, a prognostic-related model was constructed through lasso regression, which was composed of 6 miRNA genes. A risk score formula was created for each patient after integrating the expression value of each particular gene. In the lasso regression analysis, the regression coefficients estimated by this risk score formula were weighted. The median risk score value served as the demarcation point in the risk score calculation to split patients into low-risk and high-risk categories. The differences in the survival rate of the two groups were calculated using Kaplan–Meier and analyzed by log-rank statistical techniques. To investigate the accuracy of the model predictions, the survival ROC in the R package was utilized, and the C-index index was obtained using the SURVCOMP method in the R package. The prognostic model was further validated using the external dataset GSE175462. Given that the dataset did not have complete survival data, we used the support vector machine (SVM) algorithm to construct a classification model consisting of six miRNAs, and performed ROC analysis and confusion matrix visualization at the same time.

### 2.3. ceRNA Network Construction

In this study, lncRNAs that appeared at least three times were obtained from two databases, and the subsequent ceRNA network construction was carried out. The screened lncRNAs were paired with lncRNA-miRNA in the miRcode database (http://www.mircode.org). Then, mRNA prediction was performed simultaneously in the three miRNA gene prediction databases of miRDB, miRTarBase, and TargetScan. Finally, the matched lncRNA-miRNA pairing and miRNA-mRNA pairing were imported into Cytoscape (Version 3.7.2) software to construct a lncRNA-miRNA-mRNA regulatory network based on the ceRNA mechanism.

### 2.4. Prediction of RAB1A and TM9SF3 Immunohistochemistry Using the HPA Database

The Human Protein Atlas database (https://www.proteinatlas.org/), the HPA database, is a free public database of more than 26 000 antibodies targeting over 17 000 human genes. Normal tissue and LUAD tissue immunohistochemical samples were obtained by searching for the genes RAB1A and TM9SF3.

### 2.5. miRNA Target Gene Prediction and GO, KEGG Enrichment

The miRDB was used to predict miRNA target genes. Visualization was provided using the software Cytoscape. To thoroughly investigate the functional significance of these mutant genes, the R package “ClusterProfiler” was used to annotate mutant genes. To analyze associated functional categories, the Gene Ontology (GO) and Kyoto Encyclopedia of Genes and Genomes (KEGG) were utilized. The GO and KEGG enrichment pathways are considered significant, with *p*-value and *q*-value <0.05.

### 2.6. Immune Cell Infiltration Analysis

The CIBERSORT technique is used to determine the types of immune cells present in the tumor microenvironment. This approach uses the support vector regression concept to undergone convolution analysis of the expression matrix of immune cell subtypes. This includes 547 biomarkers and detects 22 human immune cell phenotypes such as T, B, plasma, and myeloid cell subsets. The expression levels of LUAD patients were checked by the CIBERSORT method, to predict the relative infiltration ratio of 22 immune cells. Following the risk score generated by the prognostic model, the subjects were classified into high-risk and low-risk groups based on the median. The difference in the level of infiltration of 21 immune cells was assessed in the high-risk category and the low-risk category (as the infiltration level predicted by T cells CD4 naive in all samples was 0, it was not included in the analysis). The *p*-value was calculated using the Mann–Whitney test method.

### 2.7. Statistical Analysis

The Kaplan–Meier analysis produced the survival curves, and the log-rank was utilized to compare and detect the *p*-value. For univariate analysis, the Cox proportional hazard model was employed and for correlation analysis, Pearson's test was utilized. The R programming language was used for all statistical analyses (version 3.6). These statistical tests were two-sided, with a statistical significance of *p* < 0.05.

## 3. Results

### 3.1. Analysis of Differentially Expressed miRNA and Screening of OS-Related Genes

**Figure 2:**
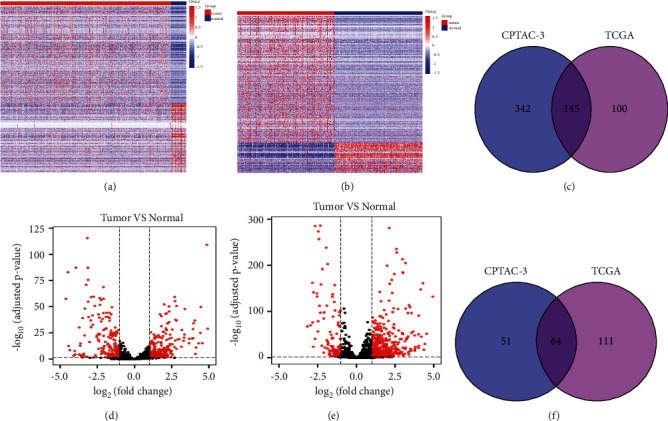
The GDC database, TCGA-LUAD and CPTAC-3, datasets were screened for 209 significantly differentially expressed candidate miRNAs. (a) Expression heatmap in TCGA; (b) expression heatmap in CPTAC-3; (c) volcano plot of the differentially expressed microRNA analysis of the TCGA data set; (d) volcano plot of the analysis of differential expression of CPTAC-3 data set. Red represents differentially expressed genes and black represents non-differentially expressed genes; (e) intersection of upregulated genes in tumors; (f) intersection of downregulated genes in tumors.

**Figure 3:**
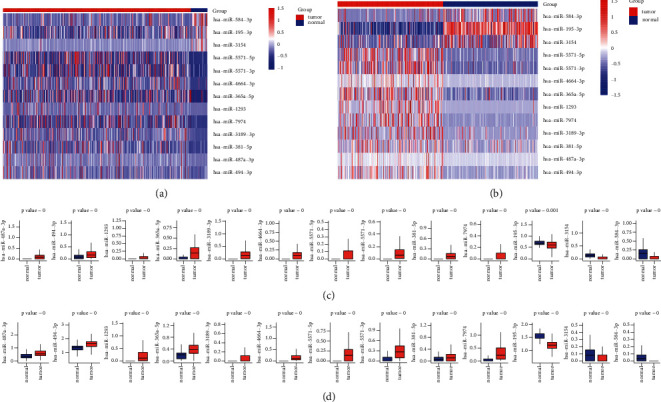
Univariate Cox regression analysis and Kaplan–Meier test on the selected differentially expressed miRNAs to further filter out eight miRNAs that are significantly related to OS. AB: 13 candidate microRNAs that are differentially expressed in tumors and normal tissues and are significantly related to OS ((a) expression heatmap in TCGA, (b) expression heatmap in CPTAC-3); CD: 13 candidate miRNAs in normal tissues (para-cancerous tissues) and tumor tissues. (c) Expression in the TCGA data set, (d) expression in the CAPAC-3 data set.

There are two publicly available LUAD tumor data in the GDC database. We downloaded the original miRNA transcript expression data from the TCGA-LUAD and CPTAC-3 datasets. The difference in expression of miRNAs that were upregulated and downregulated in the two datasets was found using differential analysis (Figures 2(a)–2(d)), respectively. Following the intersection, 145 genes were significantly upregulated in tumor tissues (Figure 2(e)) while 64 genes were significantly downregulated in tumor tissues (Figure 2(f)). Next, we performed a univariate Cox regression analysis on these 209 differentially expressed miRNA, and screened out 13 genes related to OS (Figures 3(a)–3(d)). The Kaplan–Meier test was used to further filter out 8 miRNAs significantly related to OS as the included genes for the subsequent prognostic model establishment. These 8 miRNAs were miR-494-3p, miR-1293, miR-4664-3p, miR-5571-5p, miR-5571-3p, miR-7974, miR-195-3p, and miR-584-3p, respectively.

### 3.2. Model Construction and Evaluation

**Figure 4:**
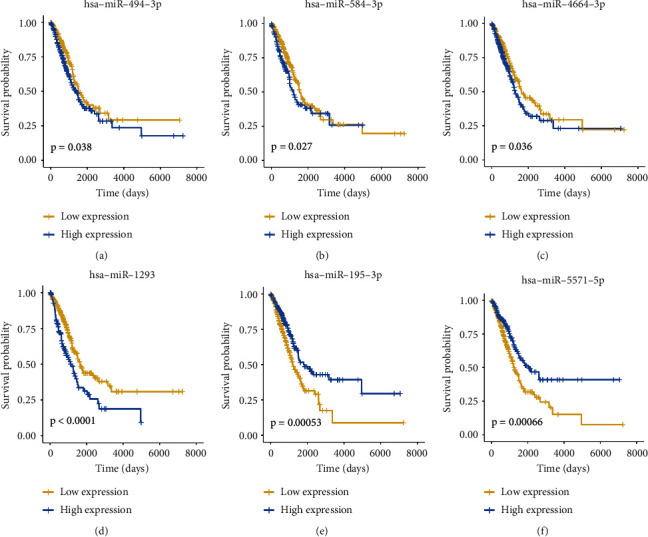
(a–f): KM survival curve of six microRNAs on OS in TCGA sample. *p* value was calculated by log-rank. The six genes can be used as independent prognostic variables for OS. Specifically, miR-1293, miR-195-3p, and miR-5571-5p were significantly related to OS. The greater the expression of miR-195-3p and miR-5571-5p, the better is the LUAD OS.(a) hsa-miR-494-3p; (b) hsa-miR-584-3p; (c) hsa-miR-4664-3p; (d) hsa-miR-1293; (e) hsa-miR-195-3p; (f) hsa-miR-5571-5p.

**Figure 5:**
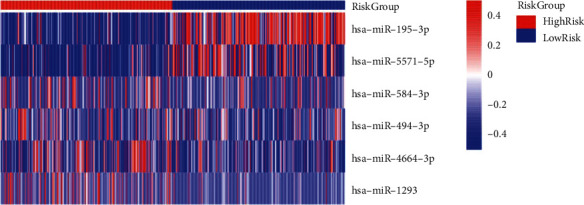
Expression heatmap of six miRNAs was used to construct the prognostic model in the high-risk group and the low-risk group.

The clinical data of TCGA-LUAD patients were gathered. A prognostic model for 8 miRNAs significantly linked to OS (*p* < 0.05) was constructed using univariate Cox regression analysis and lasso regression methods combined. TCGA patients were assigned to the training and validation groups in a 1:1 ratio at random. We utilized lasso regression analysis to get the optimal risk score value for further investigation. Finally, a prognostic model (Table 1) consisting of six genes was obtained: miR-195-3p, miR-5571-5p, miR-584-3p, miR-494-3p, miR-4664-3p, and miR-129. All six genes can be exploited as independent prognostic factors for OS. In particular, miR-1293, mir-195-3p, and mir-5571-5p were highly correlated with OS. The greater the expression of miR-195-3p and miR-5571-5p, the better the LUAD OS (Figures 4(a)–4(f)). The expression of miR-195-3p and miR-5571-5p made the most contribution to the model (Figure 5).

**Figure 6:**
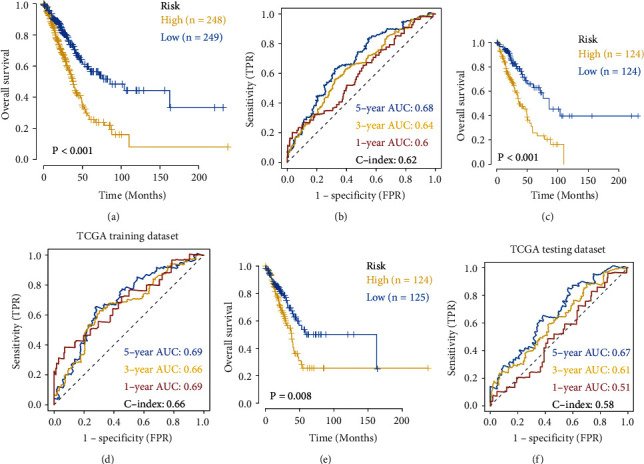
Overall survival analysis of six miRNA in training and validation sets. (a) KM analysis of the TCGA samples' OS using the risk score derived from the prognostic model. ROC evaluated the prognostic effect of the model; (c–d) the analysis in the training set; (e–f) the analysis in the validation set.

**Figure 7:**
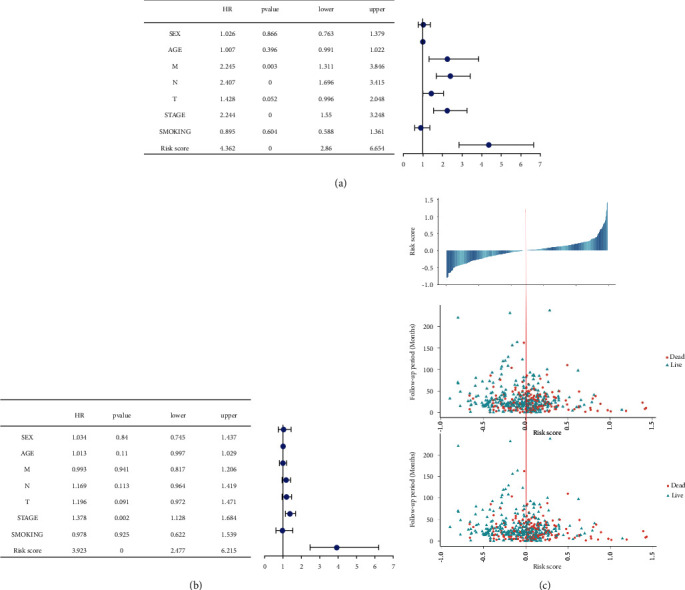
The training and validation sets' univariate and multivariate COX analyses to identify risk variables and the patient's risk survival status. (a) Univariate Cox analysis detected clinical factors related to OS; (b) multivariate Cox analysis identified clinical factors related to OS; (c) the survival time of OS (middle) and survival time of PFI (bottom) changed with the increase of the score after sorting the risk scores calculated by the prognostic model from low to high.

Based on the median risk score, the two groups of patients were high-risk and low-risk. To compare and determine the *p*-value, the Kaplan–Meier curve and log-rank were employed. In all samples, training sets, and test sets, the OS of the high-risk group was significantly lower than the low-risk group (Figures 6(a), 6(c), 6(e)). In addition, the ROC curve findings revealed that the C-index indices of all sample sets, training sets, and test sets were 0.62, 0.66, and 0.58, respectively (Figures 6(b), 6(d), 6(f)), indicating that the model had a better verification performance. The results of the support vector machine model are shown in Figure S1, the AUC of the model composed of six miRNAs reached 0.98, and the prediction accuracy rate was 95.2%, indicating that the construction of the prognosis model has good scalability. EGFR-mutated lung cancer patients receive targeted therapy with significant efficacy and prolonged survival. We wanted to investigate whether riskscore was superior to EGFR expression level as a better survival indicator for lung cancer. GEPIA (http://gepia2.cancer-pku.cn) database was used to analyze whether EGFR expression is related to survival (Figure S1C). The relationship between EGFR expression level and patient survival is not significant, indicating that riskscore is better than EGFR. To determine the prognostic value of the model, this study used univariate and multivariate COX analysis to detect the impact of gender, age, smoking or not, AJCC staging, TNM staging, and other clinical risk factors on the prognosis. According to the findings, AJCC pathological stage was considered an independent marker for prognosis of OS based on the six miRNA prognostic models (Figures 7(a) and 7(b)). After sorting the risk scores using the prognostic model from low to high, the survival time of OS and progression-free interval (PFI) changed accordingly with the increase of the score (Figure 7(c)), the higher the score, the less time the patient survived and the higher the death rate.

### 3.3. Discussion on Specific Signal Mechanism Related to the Prognosis Model

**Figure 8:**
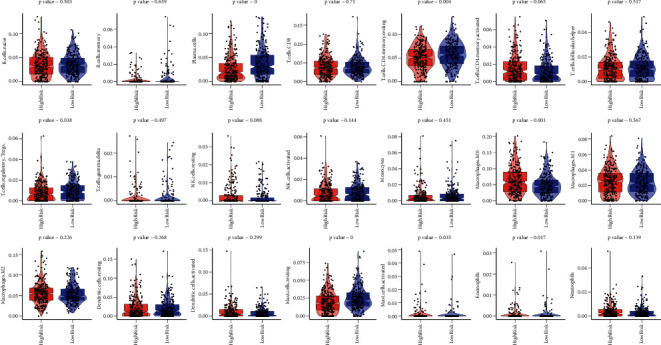
After risk scoring with a prognostic model, the high-risk and low-risk groups were defined using the median. CIBERSORT was utilized to estimate immune cell infiltration levels and comparison of cell infiltration levels in the high-risk and low-risk groups. *p* value was calculated using the Mann–Whitney test.

Tumor-related fibroblasts, immune cells, extracellular matrix, and a range of growth factors, inflammatory agents, unique physical and chemical properties, and cancer cells themselves make up the tumor microenvironment. The tumor microenvironment has a major impact on tumor diagnosis, prognosis, and clinical therapy sensitivity. CIBERSORT is the most widely used technique for determining the degree of immune cell infiltration in tumor tissues. CIBERSORT calculated the infiltration level of 22 immune cells in the LUAD sample. The probable mechanism of risk score regulating tumor immune infiltration was discovered by examining the connection between risk score and tumor immune infiltration. Following risk scoring with a prognostic model, the high-risk and low-risk groups were defined by the median. After excluding T cells CD4 naive whose predicted infiltration level was 0 in all samples, the Mann–Whitney test was used for the two groups to evaluate the difference in the infiltration level of the remaining 21 immune cells and calculate the *p*-value. These findings revealed a significant difference among T cells CD4 memory resting, macrophages M0, mast cells resting, T cells regulatory (Tregs), mast cells activated, plasma cells, and eosinophils in the high-risk group and the low-risk group (*p* < 0.05; Figure 8).

### 3.4. Construction of ceRNA Network

**Figure 9:**
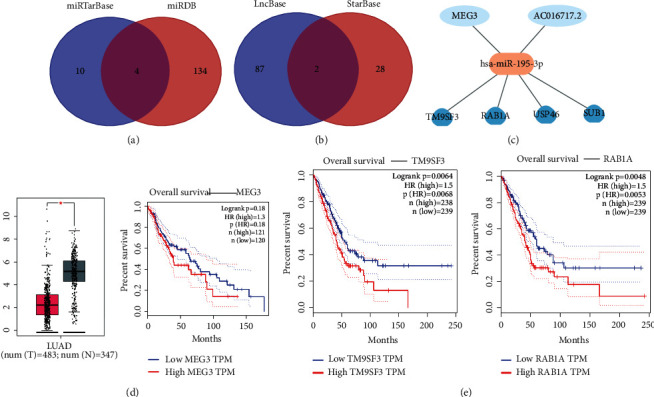
Construction of ceRNA network. (a) Results of mRNA target predicted by mirTarBase and miRDB. (b) Results of lncRNA targets predicted by lncBase predicted V.2 and StarBase. (c) The network of lncRNA-miRNA-mRNA. (d) The expression and prognostic value MEG3 in LUAD. (e) The prognosis value of mRNA RAB1A and TM9SF3 in LUAD.

A total of six miRNAs were constructed in this model, of which miR-195-3p and miR-5571-5p had the greatest contribution to the model. In this study, these two miRNAs were selected for subsequent ceRNA network construction (Figures 9(a) and 9(b)). The analysis results showed that no associated lncRNA molecules were found for miR-5571-5p, and two lncRNAs, MEG3, and AC016717.2, acted as upstream regulatory elements of miR-195-3p. Further, the miR-195-3p target genes TM9SF3, RAB1A, USP46, and SUB1 were predicted through the database, and the ceRNA network was shown in Figure 9(c). Expression profiling analysis found that MEG3 was downregulated in LUAD patients compared with normal tissues, and survival analysis showed that LUAD patients with high MEG3 expression had a poorer survival trend (Figure 9(d)). We predicted survival for all four target genes of miR-195-3p, demonstrating that patients with high expression of RAB1A and TM9SF3 have a poor prognosis (Figure 9(e)). Thus, the lncRNA MEG3/miR-195-3p/RAB1A/TM9SF3 regulatory axis may play a vital role in the progression of LUAD.

### 3.5. Validation of TM9SF3 Expression Level in HPA Database

**Figure 10:**
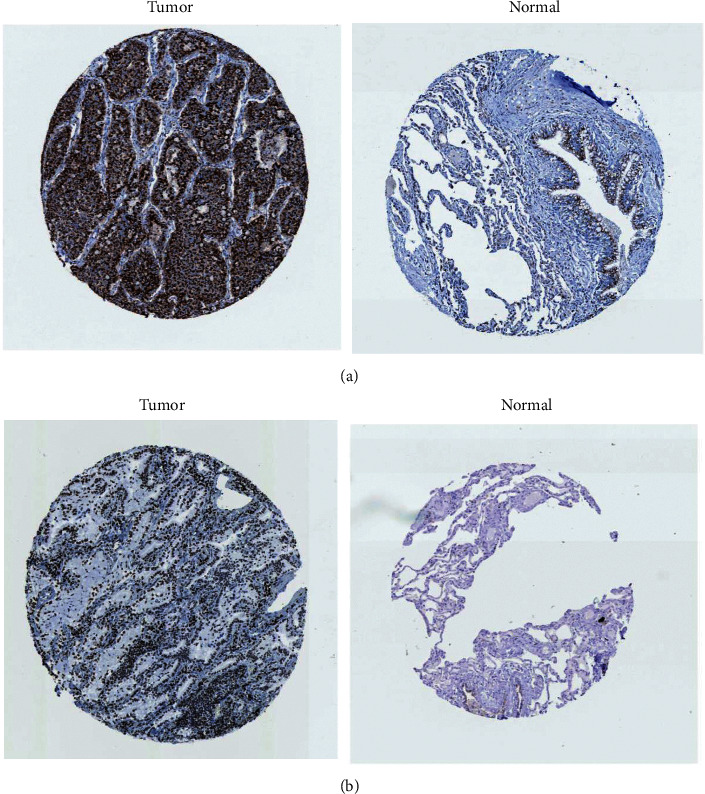
(a) Immunohistochemical staining results of TM9SF3 in LUAD from HPA database. (b) Immunohistochemical staining results of RAB1A in LUAD from HPA database.

Log-rank survival analysis was performed on the four target genes RAB1A, TM9SF3, USP46, and SUB1, and the patients with high expression of RAB1A and TM9SF3 genes had poor prognosis and low survival rate (Figure 9(e)). Further verification of RAB1A and TM9SF3, whether they are highly expressed in lung cancer patients. As shown in Figure 10, the HPA database found that TM9SF3 protein was highly expressed in LUAD tissues. The immunohistochemical staining in the HPA database showed that compared with normal tissues, the immunohistochemical staining of cancer tissues was deeper, suggesting that TM9SF3 protein was highly expressed in LUAD tissues (Figure 10(a)), however, there was no significant difference in RAB1A expression (Figure 10(b)).

### 3.6. miRNA Target Gene Prediction and GO, KEGG Enrichment

**Figure 11:**
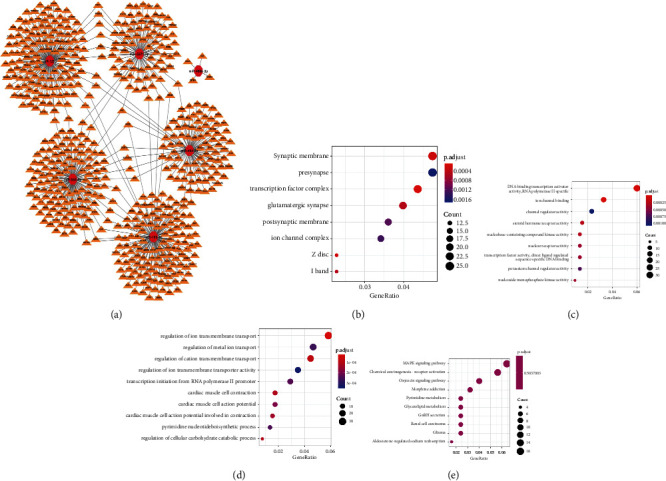
Potential connection between miRNA and target gene was visualized by Cytoscape and enrichment analysis of six target genes. (a) Visualization of six microRNA target genes; (b–d) GO enrichment of target genes (b) CC, (c) BP, (d) MF; (e) KEGG enrichment of target genes.

The miRDB database was utilized to determine the potential target genes of these six miRNAs. They were as follows: miR-195-3p:273, miR-5571-5p:162, miR-584-3p:261, miR-494-3p:188, miR-4664-3p:4, and miR-1293 : 371 (Figure 11(a)). Using Cytoscape, we visualized the possible link between miRNA and the target gene. To assess the biological activities of these target genes, we used GO enrichment and KEGG biological pathway enrichment to investigate the potential involvement of these essential miRNAs in tumor tissues (Figures 11(b)–11(e)). GO analysis revealed that the biological process (BP) of target genes was enriched in the regulation of ion transmembrane transport and the regulation of metal ion migration. Molecular function (MF) was enhanced in the DNA-binding transcription activator activity and ion channel binding, while cell components (CC) were mainly enriched in synaptic membranes, transcription factor complexes, etc. KEGG biological pathway analysis showed that the MAPK signaling pathway and chemical carcinogenesis-receptor activation pathways were enriched.

## 4. Discussion

miRNA is a highly conserved noncoding RNA with approximately 19–25 nucleotides [10]. They induce posttranscriptional silencing by specifically binding with complementary sites of the target mRNA's 3′untranslated region (UTR) [11]. miRNA is related to many biological functions, such as proliferation, development, differentiation, apoptosis, and metabolism [12]. Many studies showed that abnormal miRNA expression is significantly linked to tumorigenesis, and it is now a hot research area [13, 14]. The most prevalent subtype of lung cancer is adenocarcinoma, which has a high worldwide morbidity and mortality rate [15]. It is critical to identify precise biomarkers for it. Increasing data suggest that miRNAs have a crucial function in the prevention of LUAD [16, 17]. miRNA has been demonstrated to be a complex combination of gene expression and pathway regulatory systems, as well as prognostic markers and therapeutic targets of different cancers, including lung cancer [18]. Many miRNAs have important roles in the occurrence, progress, and metastasis of lung cancer through regulating a variety of processes, including cancer initiation and progression. Some miRNAs associated with prognostic value have been discovered thus far, such as miR-221 [19], miR-372 [20], miR-429 [21], miR-486 [22], and miR-137 [23]. However, many miRNAs are yet to be discovered in LUAD, and their roles are yet to be clarified.

Here, a genome-wide analysis of miRNAs was conducted for many LUAD patients from TCGA and CPTAC-3 and found that miR-195-3p, miR-5571-5p, miR-584-3p, miR-494-3p, miR-4664-3p, and miR-1293 were differentially expressed in cancer cells than normal cells and were significantly related to OS. Specifically, miR-1293, miR-195-3p, and miR-5571-5p have significantly correlated with soothe higher the expression of miR-195-3p and miR-5571-5p, the longer LUAD survives. Moreover, the ROC analysis results of this study indicated that the AUCs of all six miRNAs in LUAD were more than 0.6, indicating that these six miRNAs had a high diagnostic value for LUAD and may be used as LUAD biomarkers. In addition, these six miRNAs have been found to be abnormally expressed in a variety of tumors. We summarize them in Table 2.

According to a few studies, miR-195-3p is linked to the development of renal cell carcinoma, cervical cancer, and oral squamous cell carcinoma. The miR-195-3p expression is upregulated in renal cell carcinoma and decreased in other cancers [24–26]. Thus, miR-195-3p can be a possible biomarker for a variety of malignancies that can be utilized for detection, targeted treatment, or prognosis prediction. The miR-5571-5p possesses the potential to be a diagnostic biomarker for dilated cardiomyopathy and is related to NYHA classification, but there is no research on the occurrence and development of tumors [27]. Our study found for the first time that miR-5571-5p can be related to the prognosis of LUAD. As a tumor suppressor gene, miR-584-3p is related to the initiation of colon cancer, glioma, gastric cancer, renal cell carcinoma, and malignant melanoma, and is an independent prognostic factor with a good prognosis [28–32]. The miR-494-3p is upregulated in endometrial cancer, glioma, retinoblastoma, and hepatocellular carcinoma, which promotes cancer progression by regulating the PTEN/PI3K/AKT pathway [33–36]. While miR-494-3p is downregulated in synovial sarcoma, prostate cancer, osteosarcoma, and oral squamous cell carcinoma, and acts as a tumor suppressor miRNA [37–40]. Furthermore, miR-494-3p was associated with a new tumor driver of lung cancer. In NSCLC cell lines, miR-494-3p is significantly upregulated. It can improve NSCLC cell proliferation, migration, and invasion by inhibiting WT1-AS overexpression [41]. The miR-4664-3p was found to be associated with postoperative recurrence in patients with small cell carcinoma of the esophagus [42]. According to reports, miR-1293 is upregulated in pancreatic cancer and papillary renal cell carcinoma and acts as an oncogene in tumor development [42]. Moreover, miR-1293 is strongly associated with LUAD patient mortality, can be a potential biomarker for detecting LUAD prognosis, and is significantly enriched in systemic lupus erythematosus pathways [45]. This study supported the previous findings. Our results showed that miR-195-3p and miR-5571-5p were downregulated in LUAD, miR-584-3p, miR-494-3p, miR-4664-3p, and miR-1293 were upregulated in LUAD, which are potential biomarkers for LUAD.

We identified target genes and analyzed associated pathways and GO annotations to obtain insight into the molecular functions of these six miRNAs. The development and progression of lung cancer are heavily reliant on abnormal signaling pathways. It was found that these six miRNAs can regulate several key signal pathways, such as the biological process (BP) of target genes regulates ion transmembrane transport, the regulation of metal ion migration is enriched, the molecular function (MF) to DNA-transcription activator activity is enriched, and ion channel binding is enriched, while the cell component (CC) is mainly enriched in synaptic membranes and transcription factor complexes. KEGG biological pathway analysis shows that the mitogen-activated protein kinase (MAPK) signaling pathway and chemical carcinogenesis-receptor activation pathways are enriched. Furthermore, in this study, patients were grouped as high-risk and low-risk groups based on their median risk score, and the OS of all samples, training sets, and test sets in high-risk groups was considerably lower than that of low-risk groups. Ultimately, after sorting the prognostic model risk scores from low to high, we observed that the higher score had a shorter survival time and higher mortality rate. Moreover, there were significant differences in the infiltration level of 21 immune cells among the high-risk and low-risk groups.

The risk model composed of six miRNAs constructed by lasso can well assess the risk status of LUAD patients. In order to explore the biological issues behind the model, we selected miR-195-3p to construct a ceRNA network, obtained a meaningful regulatory pathway lncRNA MEG3/miR-195-3p/RAB1A/TM9SF3, and finally obtained two genes RAB1A and TM9SF3. Next, expression profiling analysis, survival analysis, and HPA immunohistochemistry found that TM9SF3 was highly expressed in the tissues of LUAD patients, and the patients had a poor prognosis. However, the RAB1A survival analysis results were not significant. Previously, we found that miR-195-3p was less expressed in LUAD compared with normal tissues. Therefore, we can speculate that miRNA-195-3p is regulated by upstream lncRNA, negatively regulates the expression of TM9SF3, and ultimately affects the occurrence and development of cancer cells in LUAD patients.

In summary, these six miRNAs are significantly associated with the survival rate of LUAD patients and may be used as potential markers for predicting the prognosis of LUAD patients. This research also has some limitations. Since the CPTAC-3 sample has fewer deaths, the survival information of this dataset is not used. Another shortcoming of this study is that only internal verification was done, without external verification. Before clinical application, more validated studies in prospective datasets are required to confirm the predictive ability of the diagnosis.

## 5. Conclusion

In this study, a prognostic model with six miRNAs characteristics was constructed based on the original miRNA transcript expression data of two public datasets TCGA-LUAD and CPTAC-3 in the GDC database, and found that miR-494-3p, miR-195-3p, miR-584-3p, miR-5571-5p, miR-1293, and miR-4664-3p, may be used as a new biomarker for prognostic prediction of LUAD. miR-195-3p in the prognostic model negatively regulates the expression of TM9SF3 through the lncRNA MEG3/miR-195-3p/RAB1A/TM9SF3 regulatory pathway. This study's findings provide new insights into the precise treatment and prognosis prediction of LUAD.

## Figures and Tables

**Table 1:** Correlation coefficients and HR of the six miRNA genes in the prognostic model.

miRNA	coef	HR	low.ci	up.ci
hsa-miR-195-3p	−0.180016151	0.882	0.814	0.957
hsa-miR-5571-5p	−0.170724292	0.476	0.284	0.8
hsa-miR-584-3p	0.07243242	1.247	1.074	1.449
hsa-miR-494-3p	0.073887821	1.058	1.004	1.115
hsa-miR-4664-3p	0.104926015	1.229	1.063	1.421
hsa-miR-1293	0.141223336	1.179	1.07	1.299

**Table 2:** The expression and role of six miRNAs in other cancers.

miRNAs	Tumor type	Effect	References
miR-195-3p	Renal cell carcinoma	miR-195-3p is upregulated in renal cell tissues and cell lines (786-O, 769P, and ACHN). Overexpression of miR-195-3p can enhance renal cell carcinoma proliferation, migration, and invasion while inhibiting cell apoptosis.	[24]
	Cervical cancer	miR-195-3p is downregulated and BCDIN3D is upregulated. miR-195-3p suppresses cervical cancer cell proliferation by targeting BCDIN3D.	[25]
	Oral squamous cell carcinoma	Downregulation of miR-195-3p increases VEGF-C expression decreases lymphangiogenesis, and therefore inhibits lymph node metastasis, reducing the risk of recurrence and metastasis in patients.	[26]

miR-5571-5p	N/A	miR-5571-5p has the potential to be a diagnostic biomarker for dilated cardiomyopathy and is related to NYHA classification, but there is no research on the occurrence and progression of tumors.	[27]

miR-584-3p	Colon cancer	The expression of miR-584-3p is negatively correlated with LOC101927746 or SSRP1 in colon cancer tissues. LOC101927746 has the potential to be utilized as a competitive endogenous RNA to inhibit miR-584-3p and activate its target gene SSRP1. The high expression of LOC101927746 indicates the poor prognosis of colon cancer patients.	[28]
	Glioma	miR-584-3p is linked to the vasculogenic mimicry (VM) of glioma cells. It is possible to inhibit the VM of malignant glioma as an effective tumor suppressor gene, which can be used as a prognostic biomarker of glioma and has potential significance for gene therapy of glioma.	[29]
	Stomach cancer	miRNA-584-3p inhibits the progression of gastric cancer by inhibiting MMP-14 expression, which is promoted by Yin Yang 1 (YY1) and is an independent prognostic factor for a good prognosis of gastric cancer.	[30]
	Renal cell carcinoma	miR-875-3p is potentially a possible biomarker for the survival of patients with renal cell carcinoma, which is linked to patient prognosis.	[31]
	Malignant melanoma	miR-584-3p significantly inhibits the mobility and growth of metformin-induced malignant melanoma cells by silencing the target genes EFEMP1 and SCAMP3.	[32]

miR-494-3p	Endometrial cancer	The expression level of miR-494-3p was significantly upregulated in EC. miR-494-3p can promote the progression of endometrial cancer by regulating the PTEN/PI3K/AKT pathway.	[33]
	Glioma	MiR-494-3p expression is considerably upregulated in neurocytoma and is inversely associated with WT1-AS expression. Co-transfection of WT1-AS and miR-494-3p reduces the activation of phosphorylated AKT (p-AKT), thereby regulating the protective immune response of malignant tumors.	[34]
	Retinoblastoma	miR-494 has the potential to act as a tumor promoter and regulate tumor progression.	[35]
	Hepatocellular carcinoma	The expression level of miR-494-3p was significantly upregulated in liver cancer tissues. It has the potential to promote the progression of hepatocellular carcinoma by regulating the PTEN/PI3K/AKT pathway.	[36]
	Synovial sarcoma	The expression of miR-494-3p is downregulated in synovial sarcoma tissues, which increases the expression of its potential target CXCR4, thereby promoting its metastasis.	[37]
	Oral Squamous Cell Carcinoma	miR-494-3p acts as a tumor suppressor miRNA in OSCC. miR-494-3p may enhance the radio-sensitivity of SAS OSCC cells and induce cell senescence.	[38]
	Osteosarcoma	The expression of miR-494-3p is downregulated in osteosarcoma tissue, and the malignant biological behavior of OS cells is promoted through the circ_0081001/miR-494-3p/BACH1 axis.	[39]
	Prostate cancer	The downregulation of miR-494-3p expression in prostate cancer may play a key role in prostate cancer through posttranscriptional regulation of CXCR4 mRNA.	[40]
	Non-small cell lung cancer	miR-494-3p is significantly upregulated in NSCLC cell lines. lncRNA WT1-AS mediates the PTEN/PI3K/AKT pathway in NSCLC through negative regulation of miR-494-3p, which inhibits cell proliferation, migration, and invasion but accelerates cell apoptosis.	[41]

miR-4664-3p	Small cell carcinoma of the esophagus	miR-4664-3p was shown to be associated with postoperative recurrence in patients with esophageal small cell carcinoma.	[42]

miR-1293	Pancreatic cancer	miR-1293 can be used as a prognostic marker for pancreatic cancer and may be involved in various cancer-related pathways, including PI3K-Akt, TGF-*β*, and pluripotent stem cell signaling pathways.	[43]
	Papillary renal cell carcinoma	Using the Cox ratio risk regression model, miR-1293 can be used as a prognostic marker for papillary renal cell carcinoma, suggesting that it may act as an oncogene in the development of tumors.	[44]
	Lung adenocarcinoma	miR-1293 is significantly related to the survival rate of LUAD patients, can be used as a new biomarker to predict the prognosis of LUAD, and is highly enriched in systemic lupus erythematosus pathways.	[45]

## Data Availability

The datasets analyzed during the current study are available from the corresponding author upon reasonable request.

## Abbreviations

NSCLC: Non-small cell carcinoma
LUAD: Lung adenocarcinoma
miRNA: microRNA
GDC: Global Data Center
TCGA: The Cancer Genome Atlas
CPTAC-3: Clinical Proteomic Tumor Analysis Consortium-3
FPKM: Fragments Per Kilobase per Million
OS: Overall survival
PFI: Progression-Free Interval
HR: Hazard ratio
KEGG: Kyoto Encyclopedia of Genes and Genomes
GO: Gene Ontology
AUC: Area under the curve
ROC: Receiver operating characteristic
BP: biological process
MF: molecular function
CC: cell component

## Supplementary Materials

Supplementary Materials Figure S1: Support vector machine model prediction of six miRNAs in GSE175462 dataset and EGFR survival analysis. A: SVM model ROC curve; B: Confusion matrix visualization; C: EGFR log-rank analysis results.

## References

[1] Mederos N., Friedlaender A., Peters S., Addeo A. (2020). Gender-specific aspects of epidemiology, molecular genetics and outcome: lung cancer. *ESMO open*.

[2] Ding Y., Zhang L., Guo L. (2020). Comparative study on the mutational profile of adenocarcinoma and squamous cell carcinoma predominant histologic subtypes in Chinese non-small cell lung cancer patients. *Thoracic Cancer*.

[3] Liu J., Yang X., Zhang L. (2020). Microarray analysis of the expression profile of immune-related gene in rapid recurrence early-stage lung adenocarcinoma. *Journal of Cancer Research and Clinical Oncology*.

[4] Hou J., Yao C. (2021). Potential prognostic biomarkers of lung adenocarcinoma based on bioinformatic analysis. *BioMed Research International*.

[5] Anna L., Gunn J., Jussi S., Rautava P., Sihvo E., Kytö V. (2021). Women have a higher resection rate for lung cancer and improved survival after surgery. *Interactive Cardiovascular and Thoracic Surgery*.

[6] García-Sancha N., Corchado-Cobos R., Pérez-Losada J., Canueto J. (2019). MicroRNA dysregulation in cutaneous squamous cell carcinoma. *International Journal of Molecular Sciences*.

[7] Zeng Z., Liu S., Cai J. (2019). miR-501 promotes hemangioma progression by targeting *HOXD10*. *American Journal of Tourism Research*.

[8] Rezaei T., Mohammad A., Zahra-Sadat H. (2020). microRNA-181 serves as a dual-role regulator in the development of human cancers. *Free Radical Biology and Medicine*.

[9] Sadakierska-Chudy A. (2020). MicroRNAs: diverse mechanisms of action and their potential applications as cancer epi-therapeutics. *Biomolecules*.

[10] Xu W.-X., Liu Z., Deng F. (2019). MiR-145: a potential biomarker of cancer migration and invasion. *American Journal of Tourism Research*.

[11] Zhang K., Zhang X., Cai Z. (2018). A novel class of microRNA-recognition elements that function only within open reading frames. *Nature Structural & Molecular Biology*.

[12] Hensley A.-P., McAlinden A. (2021). The role of microRNAs in bone development. *Bone*.

[13] Yue L., Deng X., Zeng X., Peng X. (2016). The role of mir-148a in cancer. *Journal of Cancer*.

[14] Li S., Wu Y., Zhang J., Sun H., Wang X. (2020). Role of miRNA-424 in cancers. *OncoTargets and therapy*.

[15] Wang Y., Han R., Chen Z. (2016). A transcriptional miRNA-gene network associated with lung adenocarcinoma metastasis based on the TCGA database. *Oncology Reports*.

[16] Chen L., Ren Z., Cai Y. (2021). Construction and analysis of survival-associated competing endogenous RNA network in lung adenocarcinoma. *BioMed Research International*.

[17] Wang J., Yao S., Diao Y., Geng Y., Bi Y., Liu G. (2020). miR-15b enhances the proliferation and migration of lung adenocarcinoma by targeting BCL2. *Thoracic cancer*.

[18] Zheng Q., Min S., Zhou Q. (2021). Identification of potential diagnostic and prognostic biomarkers for LUAD based on TCGA and GEO databases. *Bioscience Reports*.

[19] Guo Y., Wang G., Wang Z. (2021). Reck-Notch1 signaling mediates miR-221/222 regulation of lung cancer stem cells in NSCLC. *Frontiers in Cell and Developmental Biology*.

[20] He H., Song X., Yang Z. (2020). Upregulation of KCNQ1OT1 promotes resistance to stereotactic body radiotherapy in lung adenocarcinoma by inducing ATG5/ATG12-mediated autophagy via miR-372-3p. *Cell Death & Disease*.

[21] Guo C.-M., Liu S.-Q., Sun M.-Z. (2020). miR-429 as biomarker for diagnosis, treatment and prognosis of cancers and its potential action mechanisms: a systematic literature review. *Neoplasma*.

[22] Wang A., Zhu J., Li J. (2020). Downregulation of KIAA1199 by miR-486-5p suppresses tumorigenesis in lung cancer. *Cancer Medicine*.

[23] Xue M., Hong W., Jiang J., Zhao F., Gao X. (2020). Circular RNA circ-LDLRAD3 serves as an oncogene to promote non-small cell lung cancer progression by upregulating SLC1A5 through sponging miR-137. *RNA Biology*.

[24] Jin L., Li X., Li Y. (2017). Identification of miR-195-3p as an oncogene in RCC. *Molecular Medicine Reports*.

[25] Jin M., Wang L., Zheng T., Yu J., Sheng R., Zhu H. (2021). MiR-195-3p inhibits cell proliferation in cervical cancer by targeting BCDIN3D. *Journal of Reproductive Immunology*.

[26] Lien M.-Y., Hsiao-Chi T., An-Chen C. (2018). Chemokine CCL4 induces vascular endothelial growth factor C expression and lymphangiogenesis by miR-195-3p in oral squamous cell carcinoma. *Frontiers in Immunology*.

[27] Wang H., Chen F., Tong J. (2017). Circulating microRNAs as novel biomarkers for dilated cardiomyopathy. *Cardiology Journal*.

[28] Huang H., Cai L., Li R., Ye L., Chen Z. (2019). A novel lncRNA LOC101927746 accelerates progression of colorectal cancer via inhibiting miR-584-3p and activating SSRP1. *Biochemical and Biophysical Research Communications*.

[29] Xu S., Zhang J., Xue H. (2017). MicroRNA-584-3p reduces the vasculogenic mimicry of human glioma cells by regulating hypoxia-induced ROCK1 dependent stress fiber formation. *Neoplasma*.

[30] Zheng L., Chen Y., Ye L. (2017). miRNA-584-3p inhibits gastric cancer progression by repressing Yin Yang 1-facilitated MMP-14 expression. *Scientific Reports*.

[31] Ma C., Qin J., Zhang J., Wang X., Wu D., Li X. (2020). Construction and analysis of circular RNA molecular regulatory networks in clear cell renal cell carcinoma. *Molecular Medicine Reports*.

[32] Tseng H.-W., Li S. C., Tsai K. W. (2019). Metformin treatment suppresses melanoma cell growth and motility through modulation of microRNA expression. *Cancers*.

[33] Zhu L., Wang X., Wang T., Zhu W., Zhou X. (2019). miR-494-3p promotes the progression of endometrial cancer by regulating the PTEN/PI3K/AKT pathway. *Molecular Medicine Reports*.

[34] Qiu G., Tong W., Jiang C. (2020). Long noncoding RNA WT1-AS Inhibit cell malignancy via miR-494-3p in glioma. *Technology in cancer research & treatment*.

[35] Xu F., Liu G., Wang L., Wang X., Jin X., Bo W. (2020). miR-494 promotes progression of retinoblastoma via PTEN through PI3K/AKT signaling pathway. *Oncology Letters*.

[36] Lin H., Huang Z.-P., Liu J. (2018). MiR-494-3p promotes PI3K/AKT pathway hyperactivation and human hepatocellular carcinoma progression by targeting PTEN. *Scientific Reports*.

[37] Pazzaglia L., Serena P., Mattia V. (2019). miR-494-3p expression in synovial sarcoma: role of CXCR4 as a potential target gene. *International Journal of Oncology*.

[38] Weng J.-H., Yu C. C., Lee Y.-C., Lin C. W., Chang W. W., Kuo Y. L. (2016). miR-494-3p induces cellular senescence and enhances radiosensitivity in human oral squamous carcinoma cells. *International Journal of Molecular Sciences*.

[39] Liu S., Duan K., Zhang X. (2021). Circ_0081001 down-regulates miR-494-3p to enhance BACH1 expression and promotes osteosarcoma progression. *Aging*.

[40] Shen P.-F., Chen X. Q., Liao Y.-C. (2014). MicroRNA-494-3p targets CXCR4 to suppress the proliferation, invasion, and migration of prostate cancer. *The Prostate*.

[41] Wu C., Yang J., Li R., Lin X., Wu J., Wu J. (2021). LncRNA WT1-AS/miR-494-3p regulates cell proliferation, apoptosis, migration and invasion via PTEN/PI3K/AKT signaling pathway in non-small cell lung cancer. *OncoTargets and Therapy*.

[42] Okumura T., Shimada Y., Omura T., Hirano K., Nagata T., Tsukada K. (2015). MicroRNA profiles to predict postoperative prognosis in patients with small cell carcinoma of the esophagus. *Anticancer Research*.

[43] Shi X.-H., Li X., Zhang H. (2018). A five-microRNA signature for survival prognosis in pancreatic adenocarcinoma based on TCGA data. *Scientific Reports*.

[44] Luo W., Wang L., Luo M.-H. (2017). hsa-mir-3199-2 and hsa-mir-1293 as novel prognostic biomarkers of papillary renal cell carcinoma by COX ratio risk regression model screening. *Journal of Cellular Biochemistry*.

[45] Wang W., Liu B., Duan X. (2020). Identification of three differentially expressed miRNAs as potential biomarkers for lung adenocarcinoma prognosis. *Combinatorial Chemistry and High Throughput Screening*.

