# Supplementary material for: Six MicroRNA Prognostic Models for Overall Survival of Lung Adenocarcinoma
Source: Genet Res (Camb). 2022 Aug 27;2022:5955052. doi: 10.1155/2022/5955052 (PMC9440840; doi:10.1155/2022/5955052)
Supplement: Supplementary Materials — Figure S1: Support vector machine model prediction of six miRNAs in GSE175462 dataset and EGFR survival analysis. A: SVM model ROC curve; B: Confusion matrix visualization; C: EGFR log-rank analysis results. [file 5955052.f1.docx]

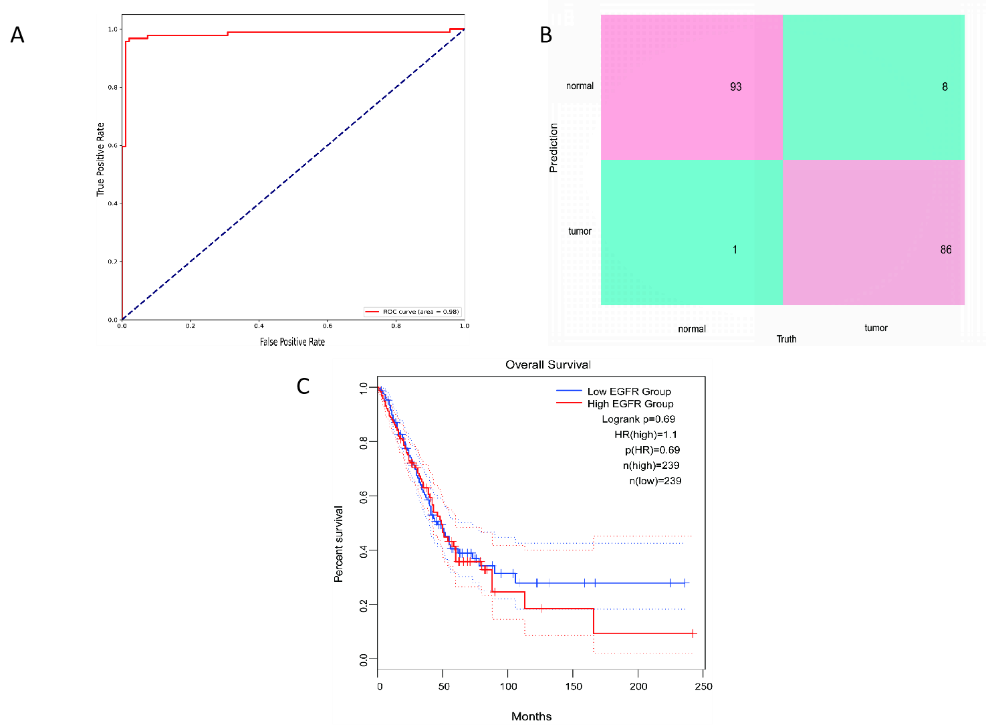


FigS1: Support vector machine model prediction of 6 miRNAs in GSE175462 dataset and EGFR survival analysis. A: SVM model ROC curve; B: Confusion matrix visualization; C: EGFR log-rank analysis results.
